# Transition from freestanding SnO_2_ nanowires to laterally aligned nanowires with a simulation-based experimental design

**DOI:** 10.3762/bjnano.11.69

**Published:** 2020-05-28

**Authors:** Jasmin-Clara Bürger, Sebastian Gutsch, Margit Zacharias

**Affiliations:** 1 Laboratory for Nanotechnology, Department of Microsystems Engineering (IMTEK), University of Freiburg, Georges-Köhler-Allee 103, 79110 Freiburg, Germany

**Keywords:** finite element method simulation, laterally aligned nanowires, planar growth, tin oxide, vapor–liquid–solid nanowire growth

## Abstract

In this study, we used simulations as a guide for experiments in order to switch freestanding nanowire growth to a laterally aligned growth mode. By means of finite element simulations, we determined that a higher volumetric flow and a reduced process pressure will result in a preferred laterally aligned nanowire growth. Furthermore, increasing the volumetric flow leads to a higher species dilution. Based on our numerical results, we were able to successfully grow laterally aligned SnO_2_ nanowires out of gold film edges and gold nanoparticles on a-plane sapphire substrates. In our experiments a horizontal 2-zone tube furnace was used. The generation of Sn gas was achieved by a carbothermal reduction of SnO_2_ powder. However, we observed no elongation of the nanowire length with an increase of the process time. Nevertheless, an alternating gas exchange between an inert gas (Ar) and an oxygen-containing process atmosphere yielded an elongation of the laterally aligned nanowires, indicating that the nanowire growth takes place in a transient period of the gas exchange.

## Introduction

Since the first reports in 1964 by Wagner and Ellis about the possibility to use a vapor–liquid–solid (VLS) process to grow semiconductor nanowires (NWs), significant work has been published on the production of nanowires [1–2]. It was demonstrated that NWs of different materials can be grown on different substrates and can be functionalized as heterostructures with the aim to understand their growth process as well as to use their unique properties in devices [2–6]. Due to their respective high surface-to-volume ratio [7], NWs are highly beneficial for recognition and manipulation of surface-sensitive processes [2,8]. As a result, Choi et al. were able to measure an improved sensitivity for gas sensors made of tin oxide nanowires (SnO_2_ NWs) in comparison with powder-based SnO_2_ thin films [9].

For the use of NWs in electronic and sensor devices, freestanding NWs often have to be scratched off of the growth substrate, diluted in a solvent and deposited on a new substrate [6,10–12]. The deposited NWs will be randomly oriented and have to be individually contacted [10,12]. Laterally aligned NWs have the advantage that no transfer from the growth substrate to a new substrate is necessary [10]. Due to their epitaxial contact with the substrate surface they are self-aligned [10]. Several methods such as template-based methods [13–14] or VLS-based methods [10,15–16] have been demonstrated in the literature. It was shown that for VLS-based methods laterally aligned NWs of different materials can be grown directly along the respective chosen substrate surface [10,15–16].

Most scientific reports on NWs focus on freestanding NWs. Especially for SnO_2_ NWs, only a few reports deal with the laterally aligned growth [15–16]. It is apparent that the process parameter space of laterally aligned NWs is even smaller than for freestanding NWs.

When the growth of laterally aligned NWs has been reported, it is commonly explained to be based on the lattice orientation of the NWs towards the crystal lattice of the substrate [15–16]. However, note that this argument is generally also used for the tilted growth of freestanding NWs towards the normal vector of the substrate surface [17–18]. Another argument for the laterally aligned NW growth mode is given by Zi et al. and is described to be the partial pressure of the process gases for the case of InAs NWs [10]. Focusing on the material system of SnO_2_, we evaluated the literature on laterally aligned SnO_2_ NWs. The growth of laterally aligned SnO_2_ NWs was reported within a temperature range between 800 °C and 900 °C with volumetric flow rates ranging from 3 × 10^2^ sccm up to 2 × 10^4^ sccm [15–16]. Furthermore, it can be concluded from a comparison of the process parameters and the data of these studies that an increase of the process pressure requires an increase of volumetric flow rate to achieve a laterally aligned NW growth [15–16]. However, a direct comparison of parameters is not possible due to differences in the experimental setups. In particular, neither the oxygen concentration nor the tube diameter were reported in the respective papers [15–16], even though these parameters will affect the growth results significantly [19–20]. Therefore, no comparison of the reported parameters using another tube furnace is feasible. Although significant parameters are missing, it can be concluded that a high process pressure implies a high volumetric flow [15–16]. This makes it necessary to analyze these influencing parameters with respect to the laterally aligned NW growth.

In this paper, we aim for an improved understanding regarding how the freestanding growth mode can be changed to the growth of laterally aligned NWs. By comparison of numerical simulations with systematic experiments, we discuss how the process parameters for freestanding SnO_2_ NWs have to be modified to achieve a laterally aligned NW growth mode.

## Experimental

### Substrate preparation

The substrates were prepared by two different preparation methods for the catalyst seeds, which are required for the VLS process. As a catalyst material, gold was chosen. First, a-plane sapphire substrates with structured gold thin films (thickness 5 nm) by metal evaporation, and second, a-plane sapphire substrates covered with gold nanoparticles (Au NPs) were prepared. For the latter method, the substrates were cleaned with deionized (DI) water and dried with nitrogen before coating them with 10 µL of a 1:2 v/v% solution of Au NPs (80 nm gold nanospheres, citrate NanoXact 0.05 mg/mL, nanoComposix) and methanol. After a rest duration of 10 min, the samples were cleaned with DI water and dried with nitrogen. The as-cleaned samples were dried in a desiccator and afterwards cleaned in a UV/ozone cleaning system to remove residual organics at the substrate surface.

### Growth and characterization of the nanowires

The NW growth was performed within a horizontal two-zone furnace by means of carbothermal reduction (further details of the complete setup are given in [19]). The use of a two-zone setup allows for an improved process control due to the individual control of the substrate and powder temperature [21]. Two-zone furnaces allow for an extended homogeneous growth even on a cm range, which is in a much longer tube section than possible in a one-zone furnace [19]. The quartz tube (Ø_inner_ = 5 cm) is attached vacuum-tightly to a gas inlet and a gas outlet. The gas inlet is connected to two mass flow controllers for individual adjustment of the introduced volumetric flow of the carrier gas, Ar, and the process gas, O_2_. The gas outlet is connected to a pumping system for base pressure evacuation and process pressure control.

The samples, which were previously covered with catalyst, were arranged in the downstream temperature zone. An alumina boat with 0.3 g of a 1:1 w/w% mixture of pulverized SnO_2_:graphite was placed in the upstream temperature zone. After closing the system, the pressure was pumped down to a vacuum level below 4.6 × 10^−5^ mbar by means of a turbo molecular pump. This step was performed to remove residual water and oxygen from the process atmosphere. To reach the respective process pressure, 40 sccm Ar was introduced into the system. After reaching the process pressure, the Ar flow was regulated to 25 sccm. For maintaining the process pressure, a membrane pump was used. Meanwhile, the tube furnace was heated up to the required process temperature (substrate temperature *T*_substrate_ = 850 °C, powder temperature *T*_powder_ = 950 °C). These temperatures are required to allow for NW growth and to supply sufficient Sn vapor from the carbothermal reduction, respectively. After reaching the process temperature, the inflow was changed to a gas mixture of 5% oxygen in argon, which initiates the NW growth. Although often no inflow of oxygen is reported in literature, oxygen as a process gas has to be provided and – as we showed previously – it cannot originate from the precursor powder [19]. The oxygen of the thermally reduced SnO_2_ powder of the precursor will be bound in the CO/CO_2_ gas generated by the carbothermal reduction. After a specified process duration, the introduced gases were changed back to 100% Ar and a volumetric flow of 25 sccm was maintained during the cool down phase the furnace passively. In the following, this procedure is called the standard procedure.

The characterization of the samples was performed using a high-resolution scanning electron microscope (SEM, Nova NanoSEM 430) from FEI.

## Results and Discussion

### Numerical results

The following section is aimed at a better understanding of the main influencing process parameters. Up to now, only a limited number of reports have focused on simulations of such systems [22–23]. The simulations are challenging because some parameters are unknown, such as the evaporation rate of the source materials during heating up and the amount of oxygen consumed by the carbon due to CO or CO_2_ formation [19–20].

As previously discussed, taking care to ensure a vacuum-tight system and a homogeneous temperature distribution for the powder precursor as well as for the substrate position, the main influencing parameters on the growth mode (freestanding or laterally aligned) of SnO_2_ NWs are the volumetric flow rate of the process and carrier gases and the process pressure of the system. These will affect the local process gas ratios at the sample site. Wang et al. and Kim et al. used a horizontal one-zone tube furnace [15–16]. The powder for the carbothermal reduction was positioned in the middle of the furnace and the samples were put downstream with respect to the powder position [15–16].

**Figure 1:**
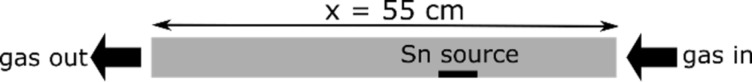
Simulation setup of the quartz tube (Ø 5 cm).

For the simulations of the tube system presented in this work, the metal vapor concentration as a function of the volumetric flow rate and the process pressure were simulated by means of the finite element method (FEM) software COMSOL multiphysics*®* [24]. Tube furnace simulations for the general growth of NWs have already been presented in literature, but have focused mainly on an improved understanding of the growth of ZnO NWs and are not specialized on the process parameters for laterally aligned NWs [22–23]. The simulation should also provide information about the influence of modification to the volumetric flow and the process pressure for the systems reported in literature [15–16]. Therefore, we simulated a one-zone furnace instead of a two-zone furnace with the powder boat within the upstream half of the quartz tube (Figure 1). Here, the tube was simulated as cylinder (Ø 5 cm) (Figure 1). The temperature of the tube was selected to be 800 °C according to the above-mentioned publications [15–16]. For reasons of simplification and to enable a better comparison with the studies of Wang et al. and Kim et al., we chose a one-zone furnace for simulation [15–16]. The temperature was not taken into account as the main influencing parameter in these simulations because the temperature range for laterally aligned NWs lies within the same temperature range as for freestanding NWs [15–17,25]. Additionally, we found that the introduction of temperature as a variable does not affect the main conclusions.

On the right side, the gas inflow (to the left) and on the left side the gas outlet were simulated (Figure 1). The inflow was set in standard cubic centimeters per minute (sccm) to be comparable to the real setup (Figure 1). The powder boat was simply simulated as a virtual inlet of Sn gas. A simulation of the carbothermal reduction itself would be difficult because of its complex dependencies [26]. However, this workaround will not affect the derived conclusions. We could not find reliable parameters for Sn diffusion, therefore, the parameters for Zn were used (*D*_n_ = 1.4 × 10^−4^ m^2^/s|_1063 K,1 atm_, adapted for the given conditions with Equation 1) [27–28]. ZnO NWs can be grown by carbothermal reduction, too [28]. Hence, the simulations do not allow for a quantitative comparison, but for a qualitative one. A metal generation of 10^−4^ mol/m^3^ by means of the carbothermal reduction was chosen according to [23].

**Figure 2:**
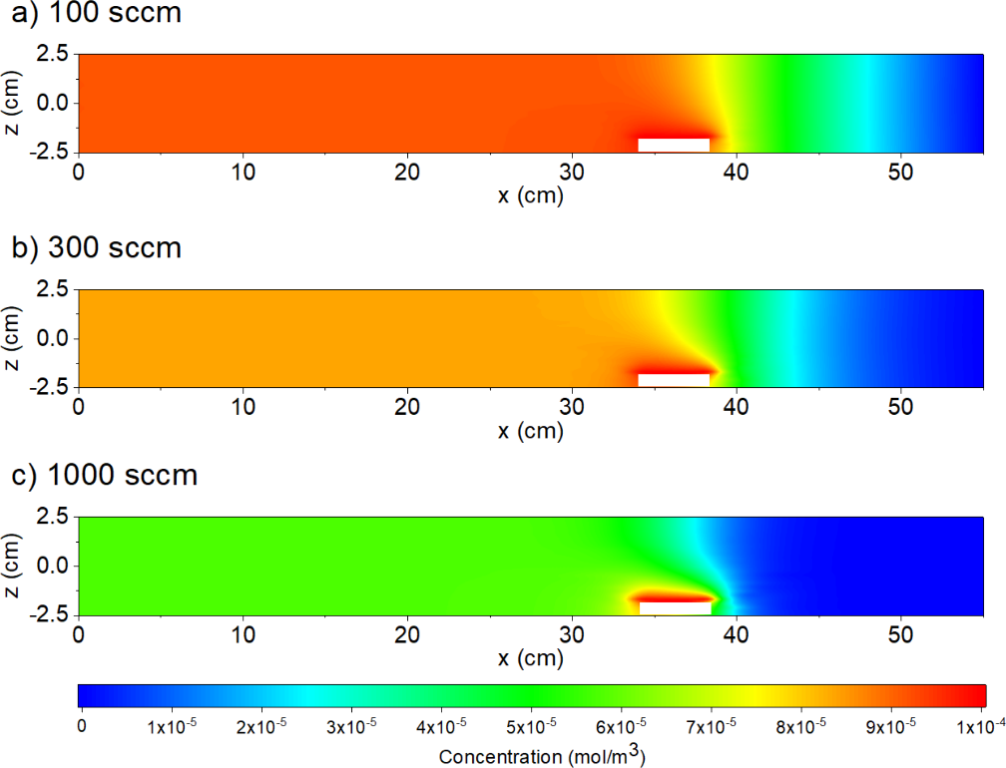
Influence of the total volumetric inflow on the stationary metal vapor concentration along the quartz tube: Simulated stationary vapor concentrations for a total volumetric inflow (from the right) of (a) 100 sccm, (b) 300 sccm and (c) 1000 sccm. The simulated pressure is 1 bar. The white rectangles in the simulations represent the powder boat. The substrates are positioned in the homogeneous downstream-oriented region.

In Figure 2 the stationary metal vapor concentration as a function of the volumetric flow is shown. Downstream with respect to the powder boat, the metal vapor concentration is homogeneous for all three simulated volumetric flows. In the upstream direction, metal vapor is found, although its concentration is significantly reduced. This is due to the two transport mechanisms of the vapor: diffusion and convection. Equation 1 presents the pressure and temperature dependence of the diffusion coefficient *D*, and Equation 2 describes the covered distance as a function of time, *t*, adapted from [28]:

[1] $$D\propto D_{n}\cdot \frac{T^{\frac{3}{2}}}{p}$$

[2] $$x_{\text{diffusion}}=2\cdot \sqrt{D\cdot t}$$

where *T* and *p* are the process temperature and the process pressure, respectively, and *D*_n_ is the tabular value of the diffusion coefficient at standard conditions.

The general transport equation is described by the following Equation 3 [28]:

[3] $$\frac{\delta \rho c}{\delta t}+\nabla \cdot \left(\rho uc\right)=\nabla \cdot \left((\rho \cdot D)\nabla c\right)+R$$

where ρ is the density, *c* is the vapor concentration, time *t*, carrier gas velocity *u*, diffusion coefficient *D* and external vapor sources *R*.

In Equation 3, the first term on the left-hand-side represents the transient behavior, the second one the convection and the first term on the right-hand-side the influence of the diffusion on the metal vapor (see Equation 4).

For the covered distance *x*_convection_ with time *t*, by convection, we get (adapted from [28]):

[4] $$x_{\text{convection}}\propto \frac{f}{A}\cdot \left(\frac{T}{p}\right)\cdot t$$

Here, *f* is the volumetric flow rate, *A* the cross-sectional area of the quartz tube and *T* and *p* are the process temperature and the process pressure, respectively.

**Figure 3:**
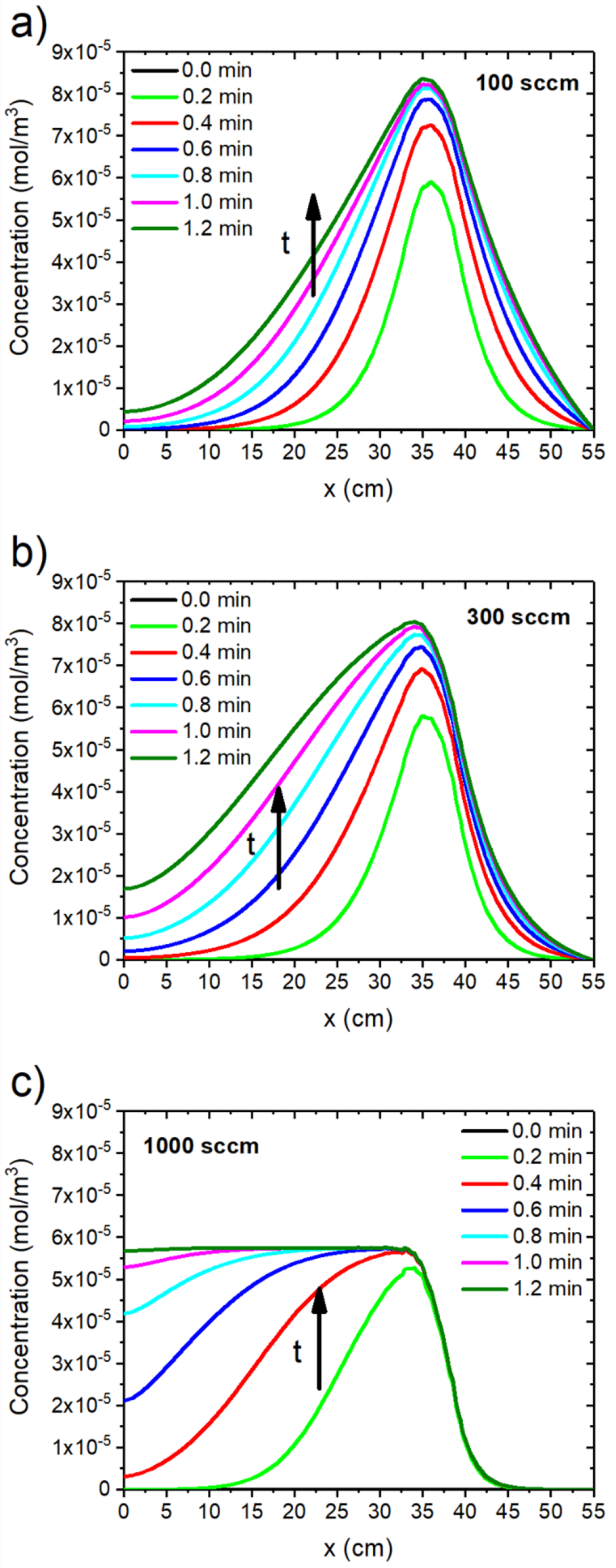
Influence of the total volumetric inflow on the development of the metal vapor concentration along the quartz tube: simulated time-resolved metal vapor concentration for a total volumetric inflow (from the right) of (a) 100 sccm, (b) 300 sccm and (c) 1000 sccm up to a time of 1.2 min. The simulated pressure is 1 bar.

**Figure 4:**
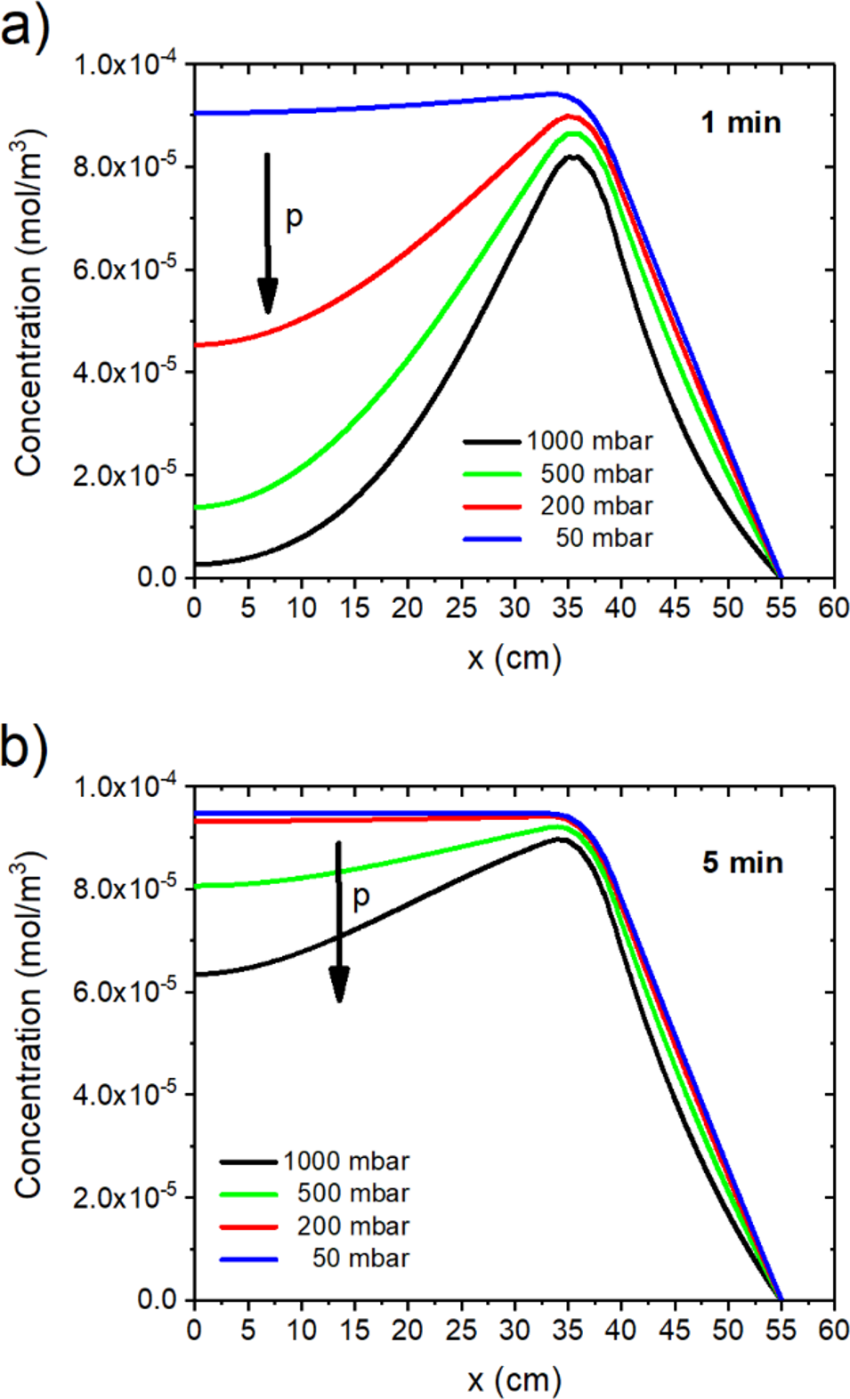
Process pressure dependence of the metal vapor concentration along the quartz tube: simulated metal vapor concentration for a variation of the simulated process pressure after (a) 1 min and (b) 5 min. The simulated volumetric flow rate (from the right) is 100 sccm.

As seen from the simulation results, the higher the volumetric flow rate per cross-sectional area, the faster the equilibrium metal vapor concentration is reached and the higher the dilution of the metal vapor (see Figure 2 and Figure 3). On the other hand, the equilibrium of the metal vapor is reached faster at a higher volumetric flow (Figure 3) and for a lower process pressure (Figure 4).

The main reason is the dependence of the metal vapor concentration on diffusion and convection [28]. The diffusion coefficient depends on the process pressure, and additionally, convection is also governed by the total incoming volumetric flow rate [28].

In case of a higher volumetric flow, the influence of the convection as compared to the diffusion increases (Figure 2 and Figure 3). For this reason, more carrier gas atoms are transported into the system within a shorter time, which results in an amplified dilution of the metal vapor that was generated by the carbothermal reduction. However, a decreased pressure will favor diffusion (Figure 4).

Hence, we conclude that an increased volumetric flow results in a faster development of the equilibrium distribution and a higher grade of dilution of the metal vapor. However, to reach equilibrium distribution faster, the selection of a decreased pressure is favorable, too. Due to technical restrictions of the used pumping systems, the upper volumetric flow rate was limited. Therefore, a reduction of the process pressure was performed in the subsequently presented experimental results.

### Experimental results

**Figure 5:**
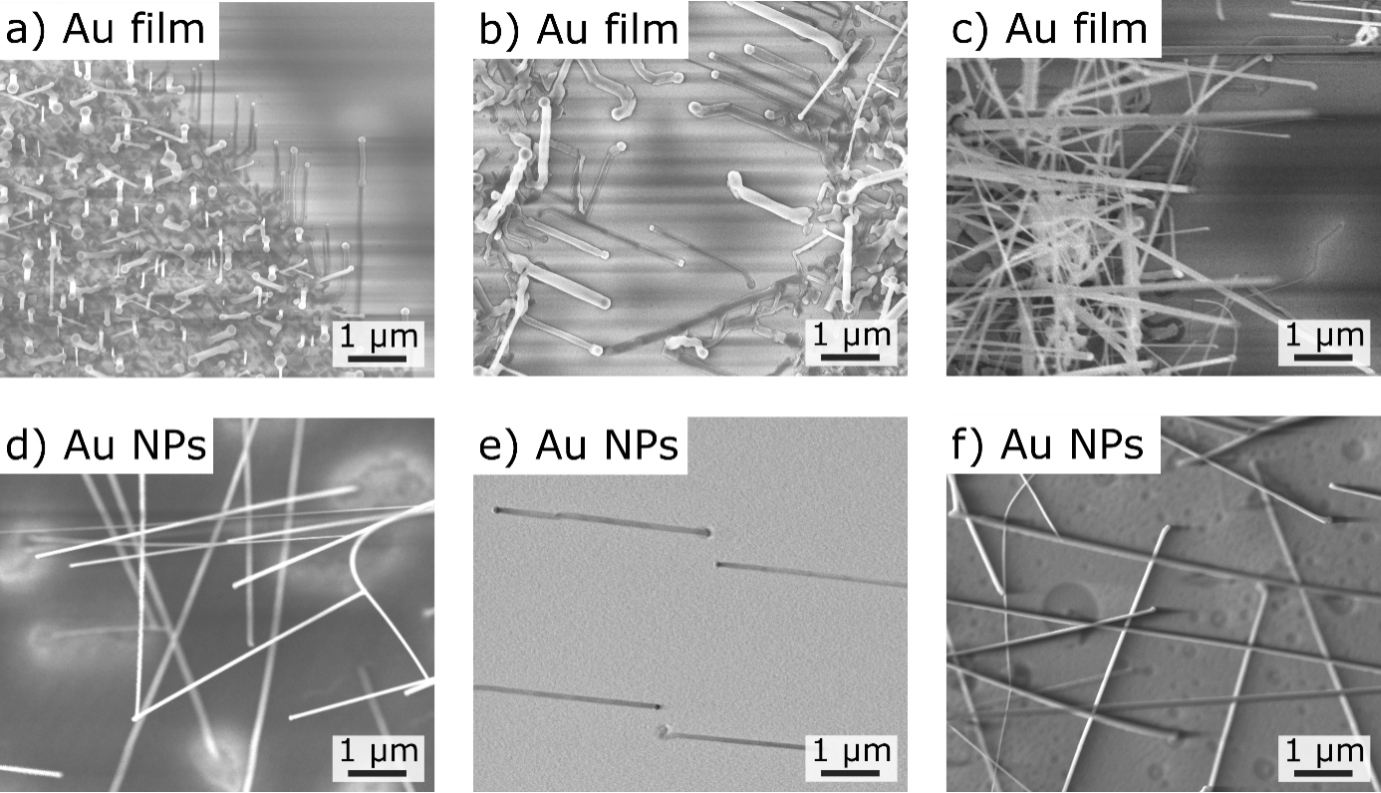
SnO_2_ NWs grown on a-plane sapphire substrates at various process pressures and powder temperatures: (a) and (d) 200 mbar and *T*_powder_ = 950 °C, (b) and (e) 20 mbar and *T*_powder_ = 950 °C and (c) and (f) 20 mbar and *T*_powder_ = 1000 °C. Gold film edges ((a), (b) and (c), film thickness 5 nm) and Au NPs ((d), (e) and (f), Ø 80 nm) were used, respectively.

In the following section we adapt our experiments based on the results from the simulations. When using the parameters of our standard procedure [19] with a process pressure of 200 mbar and an unstructured gold film, only the growth of freestanding SnO_2_ NWs is observed. As discussed above, a variation (i.e., lowering) of the process pressure is favorable and was performed starting from 200 mbar down to 20 mbar. The experimental results in Figure 5 compare the resulting NWs for two kind of samples (structured gold films, Figure 5a–c; gold nanoparticles, Figure 5d–f) for two different process pressures and powder temperatures. Especially for the nanowires from gold nanoparticles, a modification from freestanding to laterally aligned NWs is observable (Figure 5d,e). For a process pressure of 50 mbar (not shown here) or a powder temperature of 1000 °C (Figure 5c,f), a transition can be seen where the growth of the SnO_2_ nanowire begins in the laterally aligned mode and lifts up after some hundreds of nanometers to become freestanding. The growth rate is too high to maintain a quasi-thermodynamic equilibrium for the growth of straight laterally aligned NWs. In contrast, the laterally aligned NWs from gold film edges and gold nanoparticles grown at 20 mbar are highly oriented in a specific direction (Figure 5b,e).

For NWs grown out of gold films, no laterally aligned NWs are observable within the gold film; but at the gold film edge, laterally aligned NWs can be found even at 200 mbar (Figure 5a). It should be considered that for gold films or gold film edges, much higher gold droplet densities are reached than for gold nanoparticles [19,29]. The droplets compete for the tin atoms and oxygen molecules [30]. This results in a locally reduced material concentration in the surrounding area of the catalyst-decorated samples. As seen from the simulations of the volumetric flow (Figure 2), an increased volumetric flow will result in a reduced material concentration at the sample sites, thus possibly increasing the probability for laterally aligned NW growth. An increase of the powder temperature would increase the Sn gas concentration due to the endothermic characteristic of the carbothermal reduction. This results in a reduced influence of the metal vapor dilution by the process pressure.

What will be changed in the system with the reduction of the process pressure and powder temperature? A reduction of the pressure by a factor of 10 and of the powder temperature by 50 °C will result in a change in the following parameters at the sample position:

1. Ratio of the diffusion and convection: As seen from the above-presented simulation results, a reduction of the pressure will result in a higher dominance of the diffusion compared to the convection (Figure 4). The major consequence is the increased mean free path.
2. Reaction rate of the carbothermal reduction and of the NW synthesis: Not only the transport mechanisms, but also the chemical equilibria of all reactions are influenced by the process pressure and the substrate temperature. Le Chatelier’s principle describes the probability regarding in which direction a reaction occurs [31]. Hence, we would expect that an increase of the powder temperature will promote the formation of gases, which will result in an increased Sn gas and CO/CO_2_ generation (Figure 5c and 5f).
3. Local process gas concentration at the sample site: A pressure reduction will change the thermodynamic equilibrium in the surroundings of the gold droplet, which will affect the NW growth rate and their growth mode (laterally aligned or freestanding). Due to the changes in the ratio of the transport mechanisms (Equation 2 and Equation 4) and in the reaction rate, the local process gas concentrations at the sample sites are changed. Furthermore, it should be considered that the gaseous Sn atoms might already partially react with oxygen in the gas phase and that parts of the graphite, which are not used in the reaction with the SnO_2_ powder, might react with a fraction of the gaseous oxygen. An effect of these reactions is to be expected. This will result in a higher CO/CO_2_ concentration in the process atmosphere.

Summarizing these experiments, we were obviously able to transfer the NW growth from freestanding growth mode to laterally aligned NW growth by the reduction of the pressure by a factor of 10, the control of the powder temperature, and by reducing the competing Au cluster density (i.e., using a well-separated Au cluster or an abrupt gold film edge). This gives the possibility to look deeper into the laterally aligned NW growth and to achieve higher control of the resulting NW properties.

**Figure 6:**
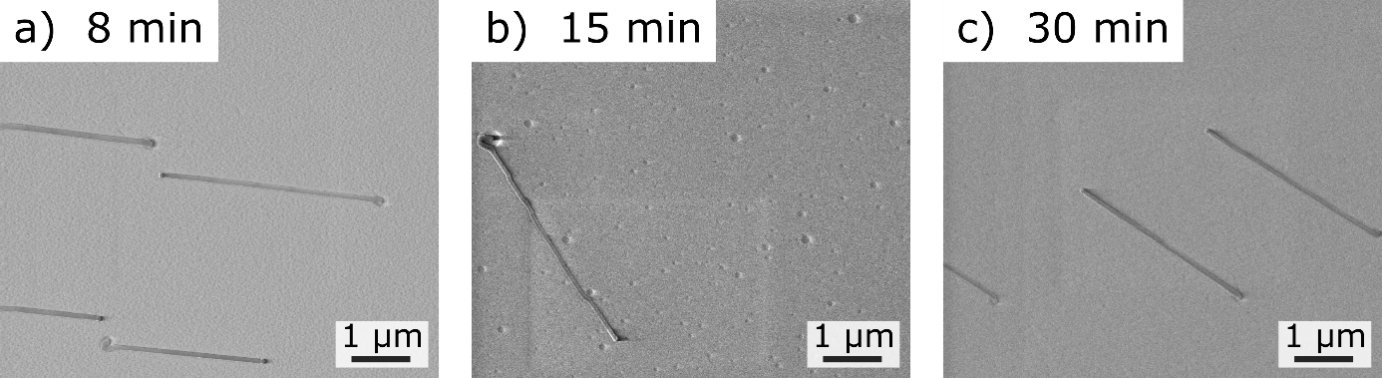
Growth experiments for an increased process time: variation of the process time from (a) 8 min, (b) 15 min and (c) 30 min. In our standard procedure, a-plane sapphire substrates and Au NPs (Ø 80 nm) were used at a process pressure of 20 mbar.

To use the laterally aligned NWs in sensor or electronic devices, control of the NW length is highly desired. Longer NWs can be contacted by means of standard lithography processes and do not require electron-beam lithography [12,28]. In the experiments presented in Figure 6, the process time was increased from 8 min to 15 min and finally to 30 min (i.e., nearly by a factor of 4).

**Figure 7:**
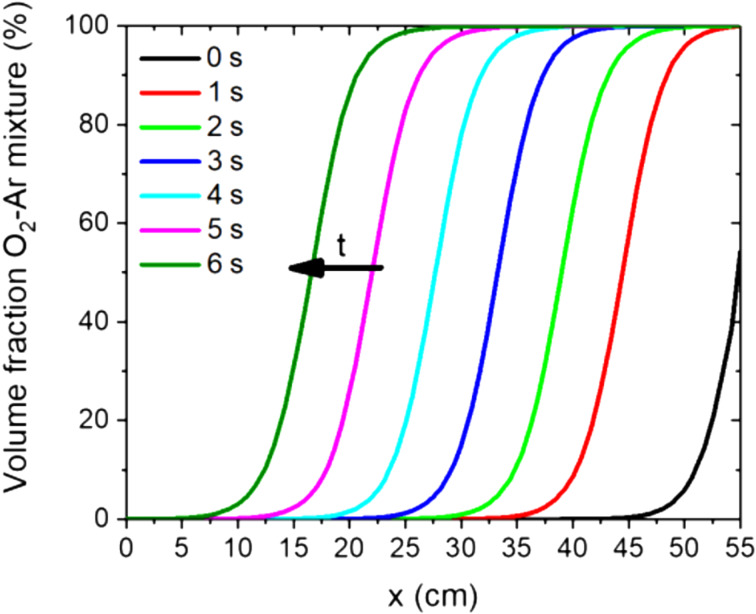
Simulated gas exchange in the quartz tube from 100% Ar to the O_2_/Ar mixture (5% O_2_ in Ar) showing the transient behavior of the gas transition: simulated total volumetric flow of 25 sccm, inner quartz tube diameter of 5 cm, pressure of 20 mbar and process temperature of 850 °C were used.

Usually the NW length can be easily increased by a longer process time [32]. Unfortunately, this could not be observed for the laterally aligned NWs as clearly seen in Figure 6. The observed laterally aligned NWs are 3.6 ± 0.3 µm long, independent of the respective process time. Therefore, we conclude that the growth of the laterally aligned NWs happens within the first 8 min of growth and then stops (Figure 6). In order to understand this behavior, the gas switching at the beginning of the process was simulated. In Figure 7, the gas switching – the change from 100% Ar to the O_2_/Ar mixture (5% O_2_ in Ar) – is shown with a total volumetric flow rate of 25 sccm, an inner quartz tube diameter of 5 cm and a pressure of 20 mbar at process temperature (850 °C). Again, the gas flow was simulated from the right side of the furnace. As expected for such systems, no abrupt gas exchange occurs (Figure 7). Instead, the gas exchange proceeds transiently, meaning that the oxygen concentration at the sample will increase continuously. These numerical and experimental results let us conclude that the laterally aligned NW growth occurs within the transient period of time and stops by reaching the equilibrium gas concentration or even before. As shown in our previous work, the growth of SnO_2_ NWs is stopped when the oxygen concentration is too high, which is due to the formation of a SnO_2_ shell [19]. The transient period of time is for all samples in Figure 6 the same – only the time of the equilibrium concentration is extended by an increase of the total process time.

**Figure 8:**
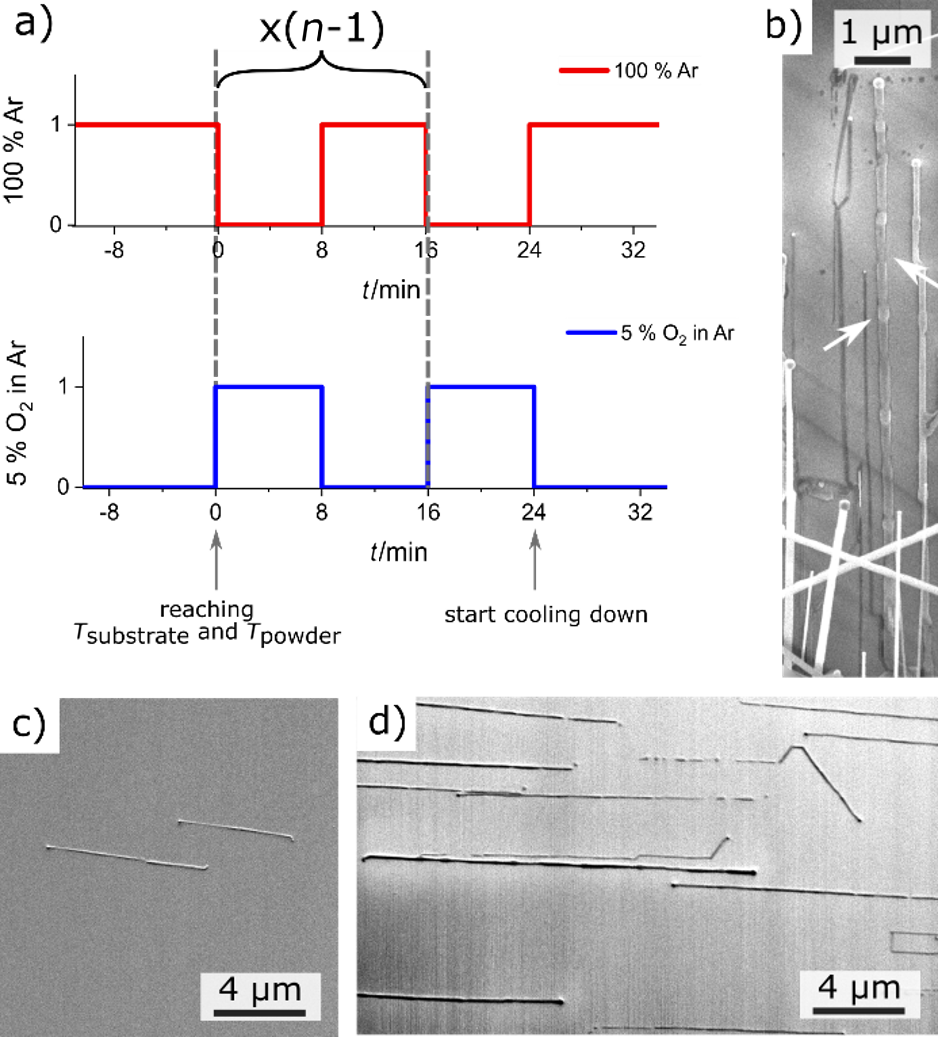
Pulsed gas inflow for the elongation of the NW length: (a) schematic diagram of the pulsed gas inflow with *n* periods and the results of the growth experiment with (b,d) 6 periods and (c) 2 periods. The residual process parameters of the growth experiments are according to our standard procedure at a process pressure of 20 mbar. In (b) a gold film edge (film thickness 5 nm) and in (c) and (d) Au NPs (Ø 80 nm) were used for an improvement of the comparability. In (a) the time *t* = 0 min corresponds to the time when the process temperature was reached.

To validate this assumption, the NW growth process was modified in the following manner by increasing the number of transient gas conditions: The gases were introduced in a pulsed manner (Figure 8a) – first 8 min of 5% oxygen in Ar, afterwards 8 min of 100% Ar, for a total period of 16 min. This 16 min cycle was repeated several times while keeping all other process parameters fixed. During the 100% Ar period of the last cycle, the process temperature is allowed to begin to cool down passively. This half step is also intuitively included in the standard procedure without pulsed gas inflow (Figure 8a).

**Figure 9:**
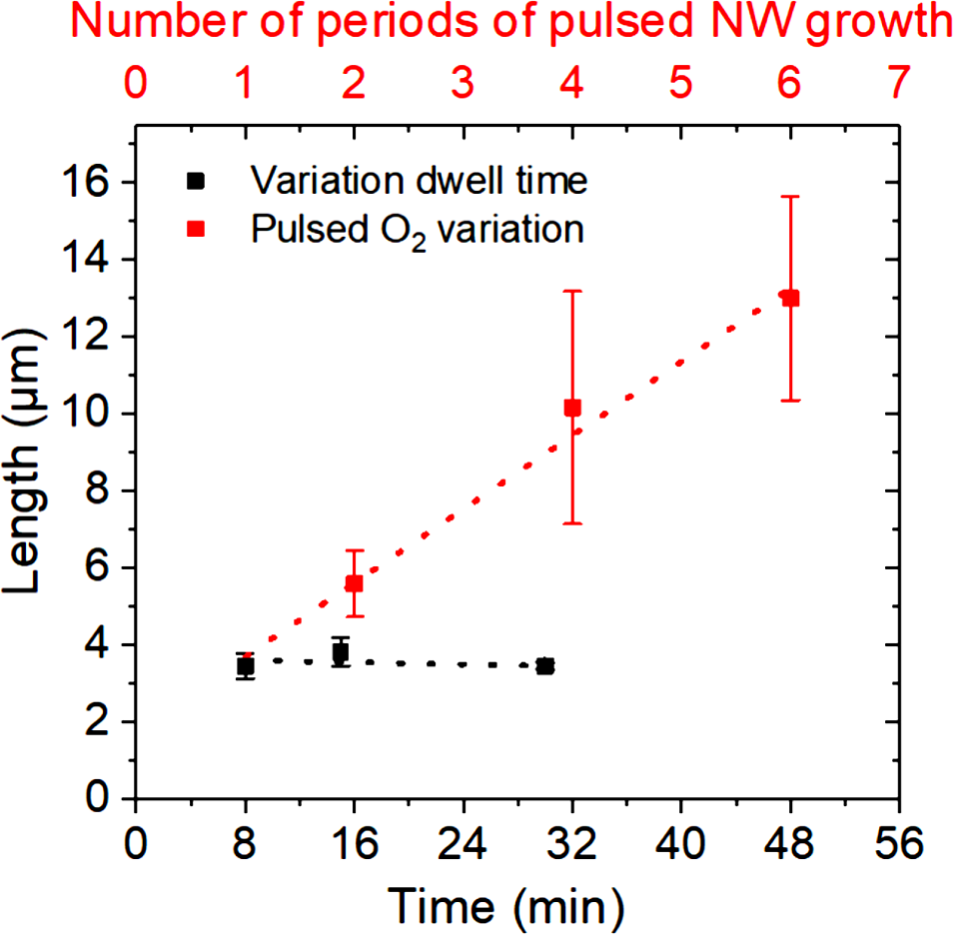
Comparison of the laterally aligned NW lengths: for different process times (black, compare to Figure 6) and for a pulsed gas inflow for a different number of repeating steps (periods) (red, compare to Figure 8) catalyzed by Au NPs (Ø 80 nm).

All NW lengths are compared for NWs grown out of Au NPs (Ø 80 nm) (Figure 8 and Figure 9). This allows the comparison of the NW lengths, which is otherwise additionally influenced by the NW diameters [33–34]. Due to the reduced volume of the smaller Au droplets, the footprint of these droplets is reduced in comparison to larger Au droplets. Therefore, for the required supersaturation and formation of one lattice plane of the smaller catalyst droplets, less material (tin, oxygen) is required. This results in an expected faster growth of thinner NWs, when the same concentration of the feeding materials in the droplet surrounding is given [19].

As can be seen from Figure 8 and summarized in Figure 9 for the evaluation of a large number of NWs (by measuring the lengths of several tens of NWs), an elongation of the NW length according to the number of repeating steps was achieved in contrast to the NWs grown under continuous oxygen supply conditions for an increased process time (Figure 9). The main cause is the multiplied transition of the transient gas conditions, in which the NW growth proceeds. This validates the assumed growth process for the elongation of the lateral growth to be still within non-stationary gas conditions. This allows for the conclusion that, also for laterally aligned SnO_2_ NWs, the ideal gas concentration lies below 5% oxygen concentration. Nevertheless, the above-presented pulsed gas inflow enables a successful increase of the NW lengths without changing the process gas concentrations.

As was shown here, by a variation of the growth time from our standard procedure, no elongation of the laterally aligned NWs was achieved. Our simulation of the gas exchange shows a transient behavior in the transition from 100% Ar to 5% O_2_ in Ar. By increasing the number of transient gas exchanges by means of a pulsed gas inflow, the lengths of the laterally aligned NWs were successfully increased according to the number of repeating steps (Figure 9).

However, the disadvantage of such a procedure is the repeated “stop” and “go” of the growth, which results in a bamboo-like laterally aligned growth with different lateral heights and diameters of the NWs (Figure 8). Especially for the thicker laterally aligned SnO_2_ NWs grown out of Au film edges, such bamboo-like structures of the NWs grown by the pulsed method are observable in the SEM images (Figure 8b). The NWs exhibit knots and in between constrictions of the NW diameter (Figure 8b). The number of knots corresponds to the number of 5% O_2_ steps (Figure 8b). An explanation is the hindrance of the NW growth by means of an oxygen concentration that is too high in the process gas atmosphere. As shown in the literature for freestanding NWs, the NW growth stops with the complete coverage and immediate oxidation of Sn at the catalyst surface in the presence of a high oxygen concentration [19]. When changing the gas atmosphere back to 100% Ar again, the transient transition between the 5% O_2_ in Ar and 0% O_2_ in Ar proceeds. The oxygen concentration decreases, and when it reaches the unknown ideal oxygen concentration, the NW begins to grow again. While the oxygen concentration is still decreasing, it will fall below the required ideal concentration for the NW growth. A sufficient oxygen concentration can no longer be supplied, and the formation rate of SnO_2_ decreases. This results in a thinning of the NW diameter. The diameter modulation of the NWs could have consequences in the electronic transport within the wires and will be analyzed in future experiments.

## Conclusion

Although SnO_2_ is highly beneficial for applications in sensors, only few systematic studies on the growth of laterally aligned SnO_2_ NWs have been published. Combining simulations with focused experiments, we were able to show that the growth of laterally aligned SnO_2_ NWs requires a decrease in process pressure and an increased volumetric flow. By means of FEM simulations, the influence of the volumetric flow and the process pressure on the metal vapor concentration in the horizontal tube furnace were analyzed. Therefore, not only the convection, but also the diffusion are contributing transport processes of the metal particles, which were generated by carbothermal reduction of SnO_2_ powder. It was proven that the metal vapor concentration at the sample site and the process pressure should be minimized. The simulations showed a faster achievement of a concentration equilibrium for higher volumetric flow rates and a reduced process pressure. Furthermore, an increased volumetric flow will result in a higher dilution of the metal particles. In summary, it can generally be stated that for laterally aligned NW growth, the overall educt concentration and the educt gradient within the system have to be minimized in total.

Building on our numerical results, we were able to tune our previous process for the growth of freestanding SnO_2_ NWs into a growth process for laterally aligned NWs. Growth experiments at structured gold film edges as well as using Au NPs (Ø 80 nm) were performed. In contrast to SnO_2_ NWs using Au NPs, a laterally aligned NW growth was observed at these edges even at a process pressure of 200 mbar. This can be explained by the locally changed process conditions in the surroundings of the dense competing Au droplets dewetted from the previously deposited gold film. By using Au NPs (Ø 80 nm), a pressure reduction by a factor of 10 was necessary to produce laterally aligned NWs. For these samples the density of the catalyst droplets is highly reduced [35] and requires a more precise adjustment of the general process conditions. This is consistent with the above-discussed necessity of a reduced educt concentration. As discussed here, the pressure reduction and the powder temperature will mainly effect:

1. the ratio of the diffusion and convection;
2. the reaction rate of the carbothermal reduction and of the NW synthesis; and
3. the local process gas concentrations at the sample sites.

This implies that the laterally aligned NW growth is thermodynamically more stable due to the interaction with the substrate. In contrast, freestanding NW growth is preferred for high incoming material rates (i.e., a higher driving force). Similar to dendrite formation in electrodeposition, the growth of freestanding NWs is promoted for high material gradients with distance from the substrate surface [36]. This can be observed for dewetted Au thin films and high incoming material rates.

Experiments were performed to continuously elongate the laterally aligned NWs by increasing the growth time. Where laterally aligned SnO_2_ NWs were successfully prepared, their length did not scale with process time. By means of a pulsed gas inflow (i.e., increasing the number of transient gas conditions), the SnO_2_ NW length was successfully elongated according to the number of repeating steps (pulses). This proves that the growth of our laterally aligned NWs occurs in the transient period of time, before reaching the gas equilibrium and after starting the oxygen inflow. The disadvantage of such transient processes is the observed bamboo-like morphology, which might influence electronic transport. Future experiments should aim to overcome this limitation, for instance, by further reduction of the oxygen concentration.
